# Design and Optimization of a Natural Medicine from *Copaifera reticulata* Ducke for Skin Wound Care

**DOI:** 10.3390/polym14214483

**Published:** 2022-10-23

**Authors:** Katieli da Silva Souza Campanholi, Ranulfo Combuca da Silva Junior, Renato Sonchini Gonçalves, Jéssica Bassi da Silva, Flávia Amanda Pedroso de Morais, Rafaela Said dos Santos, Bruno Henrique Vilsinski, Gabrielly Lorraynny Martins de Oliveira, Magali Soares dos Santos Pozza, Marcos Luciano Bruschi, Bruna Barnei Saraiva, Celso Vataru Nakamura, Wilker Caetano

**Affiliations:** 1 Chemistry Department, State University of Maringá, Maringá 87020-900, Brazil; 2 Laboratory of Chemistry of Natural Products, Department of Chemistry, Center for Exact Sciences and Technology, Federal University of Maranhão, São Luís 65080-805, Brazil; 3 Department of Pharmacy, State University of Maringá, Maringá 87020-900, Brazil; 4 Technological Innovation Laboratory in the Pharmaceuticals and Cosmetics Development, State University of Maringá, Maringá 87020-900, Brazil; 5 Chemistry Department, Institute of Exact Sciences, Federal University of Juiz de Fora, Juiz de Fora 36036-900, Brazil; 6 Department of Animal Science, State University of Maringá, Maringá 87020-900, Brazil

**Keywords:** emulgel, emulsion-filled gel, wound, phytotherapeutic, copaiba oil-resin

## Abstract

In this study, we developed a bioadhesive emulsion-filled gel containing a high amount of *Copaifera reticulata* Ducke oil-resin as a veterinary or human clinical proposal. The phytotherapeutic system had easy preparation, low cost, satisfactory healing ability, and fly repellency, making it a cost-effective clinical strategy for wound care and myiasis prevention. Mechanical, rheological, morphological, and physical stability assessments were performed. The results highlight the crosslinked nature of the gelling agent, with three-dimensional channel networks stabilizing the *Copaifera reticulata* Ducke oil-resin (CrD-Ore). The emulgel presented antimicrobial activity, satisfactory adhesion, hardness, cohesiveness, and viscosity profiles, ensuring the easy spreading of the formulation. Considering dermatological application, the oscillatory responses showed a viscoelastic performance that ensures emulgel retention at the action site, reducing the dosage frequencies. *In Vivo* evaluations were performed using a case report to treat ulcerative skin wounds aggravated by myiasis in calves and heifers, which demonstrated healing, anti-inflammatory, and repellent performance for the emulsion-filled gel. The emulgel preparation, which is low in cost, shows promise as a drug for wound therapy.

## 1. Introduction

Wounds can be defined as a discontinuity in the typical architecture of the skin, subcutaneous tissues, muscles, and bones [1,2,3]. Damage to the skin can allow microorganisms to enter, leading to inflammation and local or systemic infection. After identifying the pathological condition, therapeutic strategies must be applied quickly and accurately, reducing the morbidity, degree of contamination, size, depth of the lesion, and costs related to health recovery [1,2,3]. An inadequate, absent, or late treatment can lead to pest infestation, mainly caused by the *Cochliomyia hominivorax* fly (myiasis), which represents significant losses to public health and the global livestock industry [4,5,6].

Myiasis describes an infestation of vertebrate animals by larvae, which feed and develop as parasites [7]. Flies of *C. hominivorax*, an ectoparasite commonly found in tropical climates, are attracted to exposed skin lesions, where they can feed, reproduce, and lay their eggs [8,9]. The eggs hatch 24 h after deposition, releasing larvae that can infiltrate into lesioned, necrotic, or healthy tissues. Larval wounds rapidly assume great extension, consuming muscle and connective tissues, vessels, nerves, and cartilage. As a result, ulcerative lesions usually appear accompanied by bacterial infections (myiasis of the traumatic type, relative to open wounds), leading to death if not treated [2,10,11]. Although myiasis is a typical human disease, the problem becomes critical in cattle, sheep, horses, goats, and dogs [12,13]. In the cattle industry, it represents a significant economic impact due to falls in milk productivity (losses of millions of dollars), weight gain, and fertility index, problems that can lead small ruminants to toxemia and death. Moreover, the producer assumes high costs with treatments that generate undesirable residues in milk and meat [4,5]. These concerns encourage research into pest control methods that are effective, safe, and environmentally sustainable [14].

The wound treatment strategy is based on products that promote the elimination of infection and speed up the processes of tissue healing and regeneration [15]. Particularly, a wound associated with myiasis needs a second drug, which acts as a pesticide or antiparasitic drug [14]. However, chemical control of ectoparasites has led to the selection of resistant populations, making health management compromised and costly. Therefore, there is a growing need for research that seeks to develop alternative products that allow the simultaneous treatment of the lesion and the prevention of larval infestation, with lower costs to producers. The dermatological platform based on emulsion-filled gel has demonstrated suitable properties for treating skin lesions. These systems present adequate permanence in the skin, thereby providing physical and biological protection as well as accelerating the regeneration of the lesioned tissues [15].

Emulsion-filled gels, or emulgels, (EFGs) have been applied in the field of drug and vaccine development [16,17,18,19]. Moreover, they may be easily administered and have a pleasant texture (easy spreading on the lesion), making them a promising therapeutic technology for topical, transdermal, oral, ophthalmological, and parenteral administration [17,20,21,22]. EFGs are semi-solid biomedical systems formed by a low concentration of a gelator compound, which can be from the Carbopol^®^ and/or Pluronic^®^ class, and is responsible for immobilizing the oily components (continuous phase) in their self-assembled structures [23,24,25,26]. Carbopol 934P, for instance, produces viscous gels, once it has a high crosslinked structure. Moreover, this polymer presents excellent bioadhesive properties, and composes various formulations for dermal and ophthalmologic uses [27,28]. Pluronic^®^ F127, in turn, is a triblock copolymer of poly(ethylene oxide)–poly(propylene oxide)–poly(ethylene oxide) (PEO_106_–PPO_70_–PEO_106_) units that has been extensively used in pharmaceutical formulations [29]. It is a nontoxic, water-soluble, and biodegradable polymer with desirable thermoreversible gelation, due to its thermally induced aggregation [27,30].

*Copaifera reticulata* Ducke oil-resin (CrD-Ore) is a medicinal oil with low cost, and it may constitute a phytomedicine since it produces emulsion-filled gels. CrD-Ore is found in *Copaifera* trees, being typical from the Brazilian Northeast and the Amazon regions. Biological activities are reported for β-caryophyllene, α-copaene, β-bisabolene, hardwickiic, kovalenic, kaurenoic, polyallic, and copalic acids, existing in different amounts in copaiba oil-resin [31]. It has antitrypanosomal [32] healing effects on lesions of the oral cavity [33], larvicidal [34], antimicrobial [35,36], repellent [37], antitumor, antiblennorrhagic properties, acts as a urinary antiseptic, and ultimately heals skin diseases [38,39,40]. Studies show the effects of reducing chronic inflammatory infiltrate, edema, and specifically, the number of macrophages, with *C. reticulata* Ducke use [41]. Furthermore, copaiba oil is safe, it allows for early anti-inflammatory activity [42] and accelerated wound healing [33].

Herein, this study presents an innovative emulgel containing low polymer concentrations and a high level of *Copaifera reticulata* Ducke oil-resin (abbreviated as ECO—emulsion-filled gel with copaiba oil). While traditional emulsions are unstable and have separate phases in short periods of time, this study shows the use of optimized amounts of polymers C934P and F127, which strengthens the copaiba oil droplet interface and ensures high temporal stability. Furthermore, we have recently shown that the use of F127 favors skin permeation and releases copaiba oil in deep regions of the skin [18]. The topical dosage form acquired in this study allowed the incorporation of high concentrations of therapeutic oil-resin, ensuring significant curative and synergistic repellent benefits.

## 2. Materials and Methods

Copaiba oil-resin (*Copaifera reticulata* Ducke) was acquired by the “Copaíba da Amazônia” company (Apuí, AM, Brazil). The oil-resin was used as received. The product was registered in the National Biodiversity Authorization and Information System (SISBIO nº 72922-1) and National System for Genetic Heritage Management (SISGEN nº AE28797). Pluronic^®^ F127 (MW = 12,600 g/mol) and triethanolamine (TEA) were acquired from Sigma-Aldrich (São Paulo, SP, Brazil). Carbopol 934 (Cb) was received from Lubrizol Advanced Materials (São Paulo, SP, Brazil). Brain heart infusion broth (BHI) and Mueller–Hinton agar (MHA) were purchased from Himedia (São Paulo, SP, Brazil) and KASVI^®^ (São José dos Pinhais, PR, Brazil), respectively. All experiments were conducted using purified water obtained from a Milli-Q system (Merck Millipore, Darmstadt, HE, Germany).

### 2.1. Bioadhesive Emulsion-Filled Gel Preparation

**Scheme 1:**
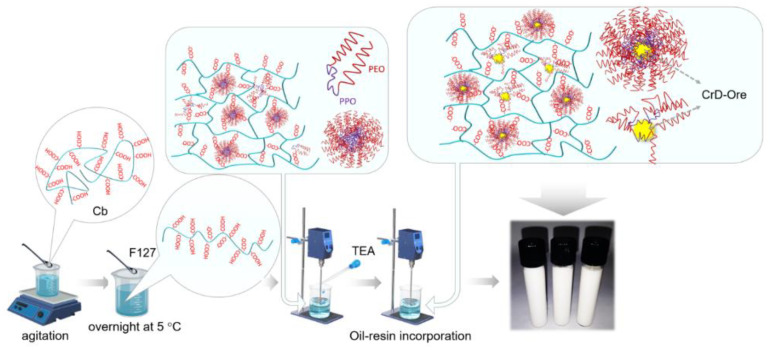
Schematic illustration of the EFG obtention.

Firstly, water and Cb were mixed until complete homogenization (Table 1 and Scheme 1). Then, F127 copolymer was added, and the system was stored overnight at 5 ± 2 °C. Next, the mixture was stirred using a mechanical stirrer (Q235-2 model, Quimis, São Paulo, SP, Brazil) (30 min, average speed of about 1000 rpm), and the pH was adjusted to 7.0 using TEA. CrD-Ore mass was slowly dripping, and the system was vigorously mechanically stirred for 30 min. After preparation, all EFG formulations were stored at room temperature for at least 24 h before each analysis. The system obtained without oil-resin (only polymers and water mixture) was called FC (F127 and Carbopol—blank system). The formulations with oil-resin were called ECO_oil concentration_ (ECO_10_, ECO_15_, and ECO_20_). The ECO abbreviation represents emulsion-filled gel with copaiba oil.

### 2.2. Morphological Characterization

The morphology of FC (without CrD-Ore) was investigated using scanning electron microscopy (SEM). First, samples were instantaneously frozen in liquid nitrogen (−196 °C) and posteriorly lyophilized for 24 h in a Thermo Micro Modulyo freeze dryer (Thermo Electron Corporation, Pittsburgh, PA, USA). Afterward, the sample was metalized in a BAL-TEC (SCD 050-Sputter Coater model, Balzers, Liechtenstein) and its morphology was evaluated (enlargements of 100 e 50 µm) in a FEI Quanta 250 microscope (Thermo Fisher Scientific, Karlsruhe, Germany) [43]. Formulations containing CrD-Ore were not evaluated due to the oil-phase mobility, which was maintained even after the dry process.

### 2.3. Accelerated Stability

This study followed the protocols of the Brazilian and European regulatory agencies [44,45]. The FC, ECO_10_, ECO_15_, and ECO_20_ were submitted to centrifugation at 3000 rpm (Centribio Co., Ltd., Shanghai, China) for 30 min at 25 °C. Then, the EFG were submitted to thermal cycles of 24 h at 5 °C, and 24 h at 45 °C, over 15 days. The physical aspects of the formulations (phase separation) were daily evaluated [46]. At least five replicates were evaluated for each EFG.

### 2.4. Texture Profile Analysis

The determination of hardness, adhesiveness, cohesiveness, elasticity, and compressibility of formulations at 25 ± 2 °C and 37 ± 2 °C were performed using a texture analyzer TAXTplus (Stable Micro Systems, Surrey, United Kingdom). The EFGs were added into a glass bottle (to avoid air bubble formation) and kept at rest for 24 h before analysis. A Delrin probe (diameter of 10 mm) was inserted twice into the emulgel to a depth of 15 mm (speed of 2 mm.s^−1^, which allowed a rest period of 15 s between the first and second compression). Measurements were performed in triplicate for each formulation.

### 2.5. Rheological Properties

Rheological properties of the EFG in continuous and oscillatory flow were performed using a HAAKE MARS II rheometer (Thermo Fisher Scientific, Karlsruhe, Germany), with parallel steel cone–plate geometry (35 mm, cone code L09006 C60/1° Ti L, separated by a gap of 0.150 mm). The rheometer is equipped with RheoWin 4.10.0000 (Haake^®^) software that allows the data fitting by rheological models, such as Casson, Herschel-Bulkley, and Power Law (Oswald-de-Waele equation) [47,48]. The models provide quantitative values for rheological parameters that enable comparisons of statistical nature. In continuous mode, the flow curves were obtained over 0 to 2000 s^−1^ of shear rate, which increases over 150 s and stays at 2000 s^−1^ for 10 s, before decreasing over 150 s. Oscillatory rheometry was performed using frequencies ranging from 0.1 to 10.0 Hz in the linear viscoelastic region (LVR), with a tension (σ) of 13 Pa. All the emulgels were analyzed in triplicate, at 25.0 and 37.0 ± 0.1 °C.

### 2.6. Microorganism and Culture Conditions

The bacterium *Staphylococcus aureus* (ATCC 25923), cultivated in BHI broth, was used. Before each experiment, the microorganism was replicated for 2 consecutive days and incubated at 37 °C for 24 h. For the tests, the cell density was standardized in tubes containing 0.9% of sterile saline solution, and the turbidity was equivalent to the reference tube (McFarland scale) which corresponds to 1 × 10^8^ colony forming unit (CFU)/mL.

### 2.7. Microbiological Analysis

Ca 1 mL of Mueller–Hinton broth and 3 g of ECO_20_ were added in each well of 12-well plates. The mixture was homogenized using a sterile tip. Then, 100 µL of the *S. aureus* suspension was added to each plate, leaving a positive control column without the dermatological platform and with the inoculum. The subculture was carried out on MHA, adding 1 mL of the mixture to a Petri dish. For the total bacterial count in CFU/mL, after incubation, pen marking was performed on the back of the Petri dishes. The experiment was carried out under aseptic conditions in a laminar flow hood. The analysis was carried out with four repetitions.

### 2.8. Statistical Analysis

The averages were compared using the free software R version 3.6.0 [49], with the RStudio interface version 1.1.463 [50]. The statistical test was applied to compare the effect of CrD-Ore on the FC properties and the temperature effect in the oscillatory rheological behavior (at representative frequencies: 0.100, 0.316, 1.000, 3.162, and 10.000 Hz), flow index, consistency index, hysteresis area, yield value, hardness, compressibility, adhesion, elasticity, and cohesiveness parameters. The normality was previously tested by the base function of the RStudio *shapiro.test ()*, and the Student’s t-test was applied by the *t.test ()* algorithm. The significance level to reject the null hypothesis was 5% (*p* < 0.05).

### 2.9. EFG Application in a Wound Aggravated by Myiasis: Case Report

This experiment used calf and heifers between 4 and 6 months of age, belonging to the experimental framework of the State University of Maringá. The animals were diagnosed with open wounds (from accidental injuries or injuries related to the dehorning of cattle). All cases were aggravated by myiasis. Before starting the treatment, the lesions were thoroughly washed with running water, and the larvae were removed with the aid of forceps. The animals were treated with CrD-Ore (n = 5, control group) and ECO_20_ gel (n = 5). The drugs (copaiba oil-resin or ECO_20_) were administered sufficiently to cover the injury and its periphery (maintaining a gel height of 1 cm above the wound). The administration of the drug was daily, until complete healing. The low number of animals is justified by the small herd of the university and by the fact that the injuries are caused by accidental causes.

## 3. Results

### 3.1. ECO: Preliminary Remarks

**Figure 1:**
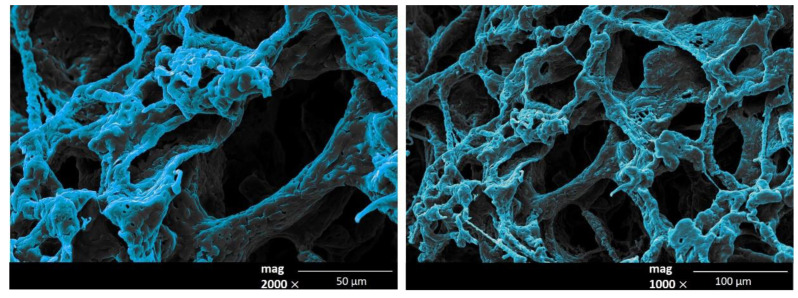
Scanning electron microscopy of the FC gel.

The emulgels presented a homogeneous appearance without lumps or precipitates. Initially, the mechanical and textural properties of the emulsion-filled gel were evaluated to predict their behavior during the manufacturing process and skin application [39,51,52]. The oil phase was incorporated into the three-dimensional crosslinked network of Carbopol (Figure 1) [28,53], with an oil drop interface reinforcement promoted by F127 and natural surfactants from the oil-resin. The presence of active phase droplets (which interact with the interfacial polymer) prevented the coalescence processes during the tests, regardless of the evaluated temperature [28]. The three-dimensional arrangement that led to the stabilizing interfaces (C934P 1.2%, *w*/*w* and F127 2.4%, *w*/*w*) of the oil-resin can be visualized in the morphological analysis images (Figure 1).

Figure 1 shows a spongy profile with thick, unconnected, and uneven porous walls that promote a three-dimensional structure. The FC morphology is attributed to the interactions between polar groups of micellar copolymer and Carbopol, consisting of crosslinking sucrose allyl esters [54]. The presence of a large superficial area and crosslinking structure of the veterinary platform justifies the accentuated drug stabilization ability and allows its release [55].

**Figure 2:**
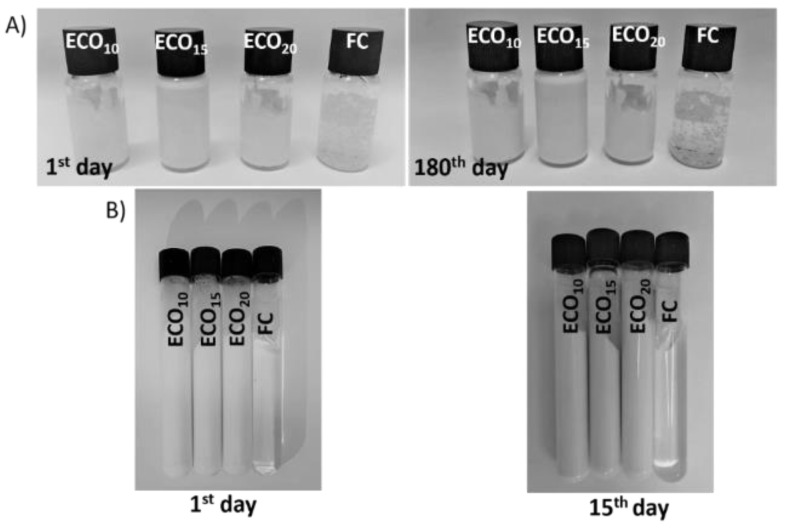
Stability of formulations: (**A**) shelf test; (**B**) cosmetic stability guide studies.

The physical integrity (phase separation or color changes) of the FC, ECO_10_, ECO_15_, and ECO_20_ systems was monitored daily, in order to study their stability. The EFGs were evaluated under storage conditions (shelf test for 180 days, performed at room temperature) and under cyclic temperatures of 5 °C (24 h) and 45 °C (24 h) for 15 days (Figure 2).

Although emulsions are thermodynamically unstable, ECO samples did not display modifications in coloration or evidence of phase separation, even under daily thermal cycles (5 °C and 45 °C, Figure 2) [56]. Additionally, shelf tests showed ECO stability up to 180 days, indicating the polymer blends provided strong interfacial properties that prevented droplet coalescence [28]. The high stability of the ECO demonstrates that it may go through thermal variations during transportation or storage without changes in its integrity. These results benefit the drug handling industry and the animal producer, since the veterinary product can be stored for prolonged periods without losing its therapeutic effects.

### 3.2. Mechanical and Rheological Properties

The textural properties of the emulsion-filled gel are displayed in Table 2. Hardness and compressibility are related to the formulation’s ability to undergo deformation over-preparation (continuous stress), package (compression during the filling process), or application steps. Cohesivity and elasticity are essential parameters for the comprehension of resistance and deformation abilities [43]. Adhesiveness simulates the work required for the formulation remotion from the original flask, predicting the formulation’s ability to be maintained on the skin for a prolonged period, therefore improving the therapeutic effect [28,57].

Most preparations followed the same trend for textural properties (Table 2). Hardness values significantly reduced (*p* < 0.05) up to 27% at 25 °C with CrD-Ore incorporation. Emollient properties can justify this behavior after oil incorporation [28]. On the other hand, variations without statistical relevance (*p* > 0.05) were observed at 37 °C for this parameter, independent of the EFG composition. The Carbopol thermo-responsiveness behavior can explain this slight variation [28]. Adhesiveness, elasticity, cohesiveness, and compressibility did not demonstrate significant variations with temperature changes and CrD-Ore presence (*p* > 0.05). These are interesting results once they revealed that high CrD-Ore presence did not impair the structure of the EFG. Thus, thermal variations and tensions in which formulations can be exposed during fabrication, filling process, transport, and skin administration, do not lead to essential changes [58]. Considering the oil-resin as the bioactive compound of the preparations, the high similarity of the EFG textural properties allowed the selection of ECO_15_ and ECO_20_ (with the highest CrD-Ore amounts) for the rheometry.

**Figure 3:**
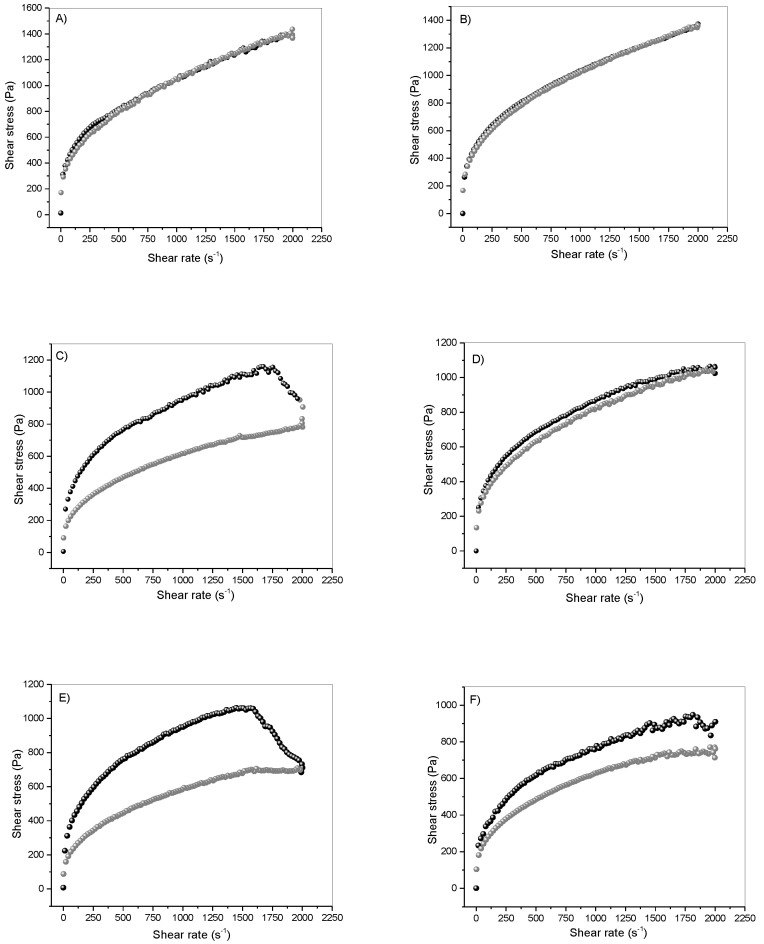
Flow curves of emulsion systems: (**A**) FC at 25.0 °C; (**B**) FC at 37.0 °C; (**C**) ECO_15_ at 25.0 °C; (**D**) ECO_15_ at 37.0 °C; (**E**) ECO_20_ at 25.0 °C; (**F**) ECO_20_ at 37.0 °C. The symbols 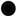 and 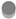 refer to upward and downward, respectively. Standard deviations are omitted for clarity; however, the relative standard deviation of the replicate analysis was less than 10% in all cases.

The rheological analysis (Figure 3) investigated the relationship between the tangential stress and the frictional movement (or velocity gradient developed in EFG) [59].

The flow curves presented in Figure 3 display the rheological profile of EFG submitted to different strains. The behavior of upward and downward curves reports two crucial characteristics for formulations: the force necessary to enable the emulgels for shearing in lamellar layers and their recovery capacity after the end of applied tension [43]. For statistical comparison, upward curves were adjusted by the Ostwald-de-Waele (Power Law) model. In this process, *K* (consistency index) and *n* (flow behavior index) parameters were obtained (Table 3).

The flow curves with a nonlinear profile (Figure 3) demonstrate a non-Newtonian pseudoplastic behavior at both temperatures (25 and 37 °C). Values of *n* were lower than the unity (Table 3), confirming this characteristic. Moreover, Table 3 displays *K* and *n* values similar to commercial dermatological emulgels reported by Osmałek and collaborators (2017) [57]. This observation means that the formulations developed here respect the rheological criteria that ensure potential acceptance by patients. The area between upward and downward curves (Figure 3) showed hysteresis occurrence and thixotropic behavior, which is related to the time-dependent variations in viscosity when shear stress is applied [60]. At the molecular level, these variations are associated with the disruption of the polymer chain interactions with the alignment effect caused by shear stress. When the shear stress ceases, the EFG recovers its previously entangled configuration [61] in a time-dependent process. The hysteresis area was more significant at 25 °C (up to a 16-fold increase, when comparing FC with ECO_20_) than at 37 °C (up to a 10-fold increase, when comparing FC with ECO_20_). This set of observations proposes a restructuration in the polymeric network caused by oil droplets, requiring more time for the initial recovery structuration.

Both FC and ECO_15-20_ emulgels (Figure 3) displayed pseudoplastic behavior visualized by a nonlinear viscosity decreasing with shear rates. This nonlinear behavior is caused by disordered and intertwined polymeric chains, which constitute pseudoplastic fluids of more excellent viscosity at rest. However, when the stress starts, it becomes ordered, and viscosity decreases [43,61,62]. The ECO_15-20_ emulgels, at 25 °C and 37 °C, showed a distinct behavior at high shear rate conditions, justified by the viscosity thermal dependence of Carbopol [63]. Although the viscosity of oils reduces with increasing temperature [28], the structuring of the carbomer under the oil effect allows a substantial increase in ECO viscosity. The high polymeric chain mobility at 25 °C facilitated the ordering of the oil droplets, which led to additional shear stress reduction. This decrease is mainly attributed to the influence of the oil droplets, since the FC gel did not show this behavior. For topical applications, pseudoplastic behavior is advantageous because the apparent viscosity can be reduced with the tension produced during manufacture or administration, making it easier to prepare or administer to the wound. Once the deformation stops, the viscosity of the ECO increases back to the original state, improving their residence time on the wound [43,62,64].

The yield value, in turn, indicates the initial stress required for the formulation to start to flow in layers, and it characterizes the flow as a nonlinear plastic type. The values for ECO emulgels were lower than the FC gel, regardless of the temperature (Table 3). These data indicate the intensity that the force must achieve to overcome the beginning of deformation, and how the droplets affect it. Moreover, the lower viscosity of the oil-resin increases the emollience of the systems and contributes to the fluidity of the formulation, as observed in the hardness values (Table 2) and yield values of the emulgels (Table 3).

**Figure 4:**
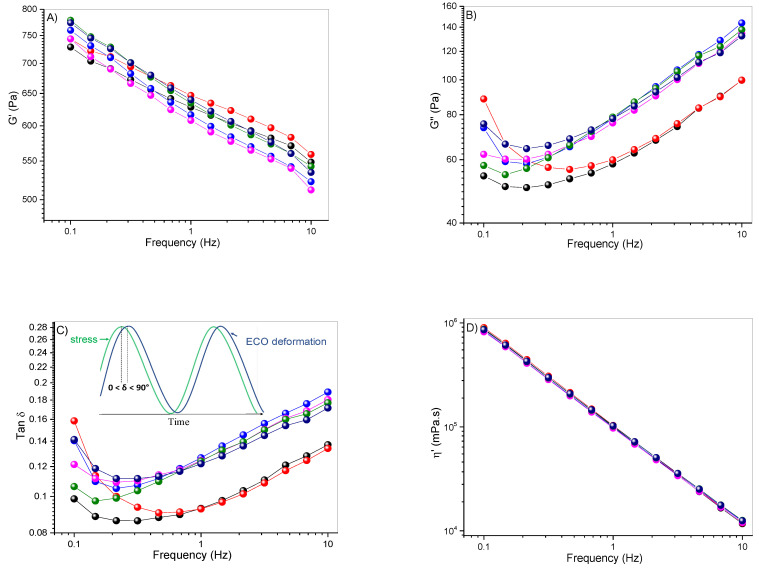
Viscoelastic properties of emulsion-filled gels in terms of oscillatory frequency: (**A**) elastic moduli (G’); (**B**) viscous moduli (G”); (**C**) loss tangent (Tan δ); (**D**) dynamic viscosity (η’). The symbols refer to 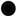 FC at 25.0 °C, 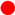 FC at 37.0 °C, 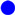 ECO_15_ at 25.0 °C, 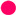 ECO_15_ at 37.0 °C, 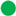 ECO_20_ at 25.0 °C, 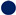 ECO_20_ at 37.0 °C. The insert (**C**) corresponds to the rheological dynamics required to obtain the lag angle.

Evaluations involving the oscillatory analysis were performed to predict ECO behavior when exposed to different natural body/skin movement stresses. Elastic (G’) and viscous (G”) modulus refer to the energy storage capacity and the dissipated energy quantity (by chains movements/relaxations), respectively. These components describe the viscoelasticity of the ECO sample and the oil-resin incorporation effect. The polymer blend is subjected to cyclic (sinusoidal) stresses, resulting in sinusoidal ECO deformations. The ECO strain and the applied stress can be compared, and the lag angle provides information regarding the structuring of the system. In this way, lag angles between 0 ≤ δ ≤ 90° provide a viscoelasticity property to the system (insert Figure 4C), with δ equal to the relationship G”/G’ [65]. The oscillatory profiles for EFG systems are presented in Figure 4.

Figure 4A displays the G’ decrease with oscillatory frequency for all systems. Significant statistical variation (*p* < 0.05) was observed at 25 °C when FC and ECO gels were compared at higher oscillatory frequencies. On the other hand, significant variations were obtained at 37 °C with FC and ECO comparison (*p* < 0.05) at lower oscillatory frequencies. These behaviors may indicate that ECO formulates maintain their G’ modulus integrity during their shelf life. According to Figure 4B, all systems displayed a significant increase (*p* < 0.05) in G” as the oscillatory frequency ranged from 0.6 to 10 Hz. No statistical relevance was observed for G” with temperature changes (*p* > 0.05).

The loss of the tangent reflects (Figure 4C) the elastic and viscous relationship of the system, which means the ability of the material to absorb and store energy. When the viscous behavior is predominant, G” > G’, the formulation demonstrated elastoviscous properties. On the other hand, systems described by low Tan δ values display the elastic nature that exceeds the viscous behavior (G’ > G”), therefore presenting viscoelastic behavior. FC and ECO samples showed lag angles lower than 1°, which characterizes the system as viscoelastic. This is in good agreement with other semi-solid systems for topical administration [28,43,64,66]. Additionally, regardless of emulgel composition or temperature, the dynamic viscosity values showed similar behavior (Figure 4D) in the studied oscillatory range. In all cases, when the oscillatory frequency increased, the η’ reduced, which corroborates with the pseudoplastic properties seen in the continuous rheology studies. This characteristic facilitates the filling, ease gel expelling from the stock bottle, and the spreadability of the formulation on the skin.

### 3.3. In Vitro Analysis of the Emulgel

**Figure 5:**
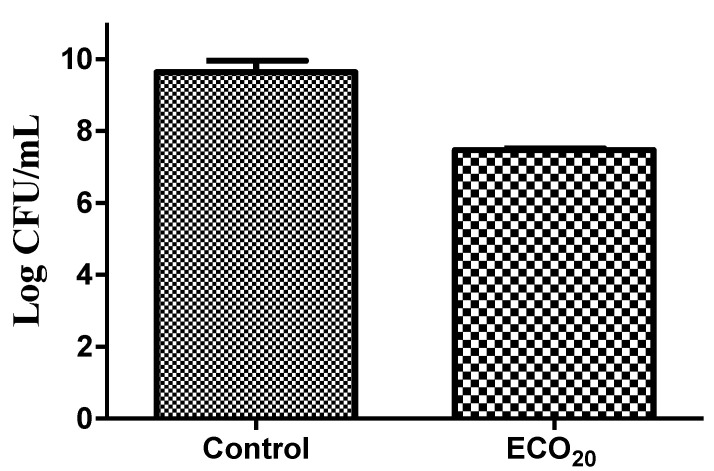
Total *S. aureus* count expressed in log CFU/mL.

The tests were performed for ECO_20_, selected by mechanical and rheological tests. The evaluations against *S. aureus* bacteria showed a significant reduction (*p* < 0.05) in the total count of microorganisms compared to the control (Figure 5). Moreover, the cells remained viable in the positive control and the presence of only F127/C934P.

The ECO_20_ presented a total count of *S. aureus* of log 7.46 CFU/mL, showing a reduction of two logarithmic units compared to the positive control, which exhibited a count of log 9.62 CFU/mL. This result shows the potential of the phytotherapeutic gel to minimize secondary infections.

### 3.4. Wound Treatment Aggravated by Myiasis

ECO_20_ and raw oil-resin (CrD-Ore—control) were selected for *in vivo* studies and used to treat two different groups. Animals diagnosed with myiasis, a pathological condition in which larvae of holometabolic insects infest ulcerative lesions, received daily topical administration of the products (CrD-Ore or ECO_20_) [12]. In the sector’s routine, conventional treatments for lesions were carried out with the use of *Boi Forte* brand spray *Mata bicheiras*, associated with Tanicid (healing powder) for complete healing. The treatments last from 15 to 30 days. However, commercial products have disadvantages related to animal productivity, since they generate residues in milk and meat. In this study, the control treatment was performed with pure oil, since its use is widely reported in skin treatment [39,67,68,69,70,71].

**Figure 6:**
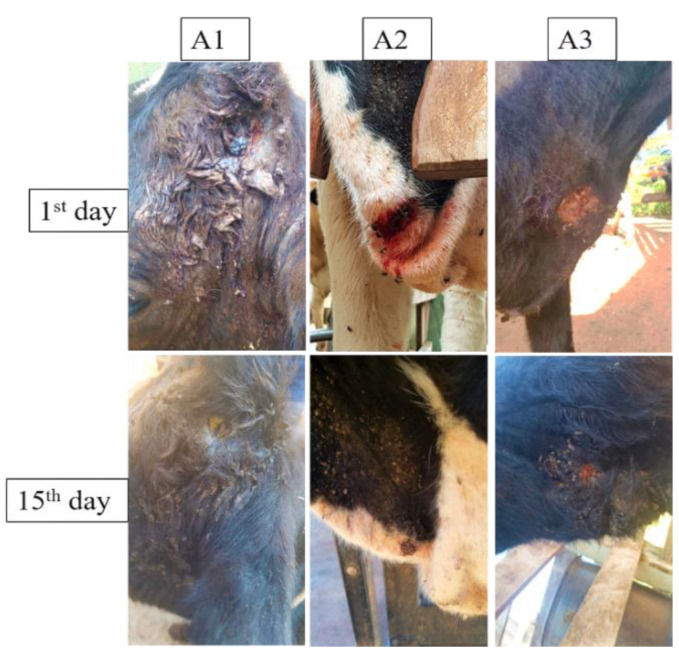
Monitoring photographs of animals treated (A1–3) with CrD-Ore. The pictures show deep lesions in the head region (horn removal wound in cattle—A1) and chest (accidental lesions—A2 and A3).

ECO_20_ formulation does not produce residues in milk and meat, making it an extremely promising therapeutic strategy for veterinarians. The healing, anti-inflammatory, and bactericidal properties of copaiba oil-resin [51,52,72,73] were evaluated, and are outlined in Figure 6. Additionally, the preparations were assessed regarding their repellent ability [37], in order to avoid a recurrence of myiasis after the initiation of the treatment.

Previously, the A1 and A3 animals showed lesions with visible signs of bacterial infection, evidenced by greenish-yellow secretions and wound odor, typical of necrotic tissue or bacterial colonization [74,75]. Initially, many flies were observed surrounding the abscess of the injured animal tissue. After the administration of CrD-Ore on all animals’ wounds and peripheral regions, there was a marked repellent effect [37]. This effect was maintained until the second application, but with less intensity. The eggs of flies deposited in the lesions were eliminated by reapplying the CrD-Ore, which interrupted the cycle of hatching and infestation, therefore indicating intense action of CrD-Ore as a larvicide [76]. The anti-inflammatory and healing properties were immediately observed by tissue regeneration in the first week of administration. At the beginning, the effects of vasoconstriction and platelet adhesion (in addition to fibrin protein) were triggered in response to the inflammatory state [75]. Moreover, the wound inflammation (first treatment day) was significantly reduced due to the CrD-Ore anti-inflammatory properties. The treatment with CrD-Ore required 15 days of application. Animals belonging to the same treatment group showed similar responses.

The oil administration was particularly difficult, due to its relatively low viscosity and easily flow from the wound, mainly with exudate presence. Therefore, the ECO formulations have the advantage of improving the residence time of the preparation on the exposed surface, with increased viscosity and adhesive properties, as observed by mechanical and rheological assessment.

**Figure 7:**
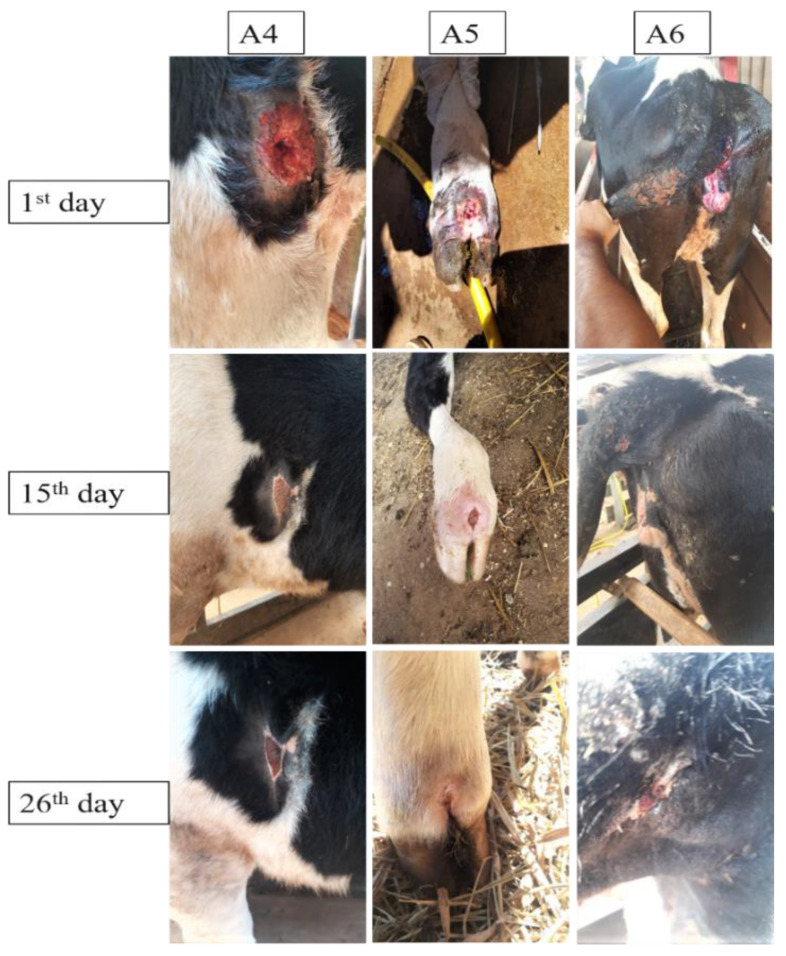
Monitoring photographs of animals treated (A4–6) with ECO_20_. The pictures show lesions in the back lumbar region (accidental injury—A4), the rear (accidental injury—A5), and in the tail insertion (accidental injury—A6).

The animals (Figure 7) showed signs of wound infection. The A4 animal presented an abscess on the wound (before treatment). The A5 animal exhibited secretions typically observed for bacterial infections, and the A6 injury showed a constant bleeding wound. The ECO_20_ offered adequate adhesiveness when applied, forming a barrier with improved thickness. Possibly, it allowed gas exchange and acted as a protective film, constraining several microorganisms. Additionally, the softness of the EFG allowed pain-free administration to the animal. The repellent effect of ECO_20_ was also observed [37], which avoided larval infestation and the progression of the myiasis injury. Indications of a curative effect were observed in the first week of treatment. After 15 days of administration, the lesions of A4 and A6 animals were completely dried. Notably, the A5 animal showed significant improvement after 20 days of administration, since the wound was placed in a region of constant movement and frequent friction. Although there were tangible signs of progress after 15–20 days of administration, the ECO_20_ was administered up to day 26 to ensure full recovery and strength of the new tissue.

According to the literature, β-caryophyllene is the main compound of copaiba oil-resin with healing effects, thus exhibiting anti-inflammatory, antibacterial, and antifungal properties. However, anti-inflammatory and analgesic effects have also been attributed to β-bisabolene, while the larvicide effect is often conferred to α-pinene and β-caryophyllene [68,76,77]. In addition, the oil-resin repellent property observed here has also been reported in other studies [70,78,79] and attributed mainly to volatile components [80]. Overall, both CrD-Ore and ECO_20_ preparations showed healing, antiseptic, larvicidal, and repellent capacity.

ECO_20_ and CrD-Ore systems showed good performance as healing and repellent systems. The ECO_20_ (20% w/w of oil concentration) showed improved properties compared to oil-resin administration, as well as relatively low cost compared to the raw copaiba oil-resin. Furthermore, ECO treatment allowed excellent topical residence and essential healing effect at the site. All these characteristics are advantageous for medical and veterinary use in economic terms.

## 4. Conclusions

The emulsion-filled gels showed high potential for veterinary applications. In addition, they displayed bioadhesive, thixotropic, pseudoplastic, and viscoelastic properties, which promoted improved retention at the wound region. Preliminary physicochemical stability studies prove the absence of phase separation, even under critical storage conditions, which aid the assurance of pharmacological activity. The *in vivo* study results show high healing, anti-inflammatory, and fly repellent capacity, verified by the absence of larvae after the beginning of the treatment with ECO and copaiba oil-resin. This behavior shows that ECO could treat the lesions aggravated by myiasis. These results represent a potential alternative for animal treatment, and can be expanded to human clinical trials.

## Figures and Tables

**Table 1:** Composition for 100 g of formulation.

Compositions		Phytotherapeutic Gels (g)		
	FC	ECO_10_	ECO_15_	ECO_20_
Carbopol	1.2	1.2	1.2	1.2
F127	2.4	2.4	2.4	2.4
CrD-Ore	0	10	15	20
Purified water	96.4	86.4	81.4	76.4

**Table 2:** Mechanical textural properties (hardness, compressibility, adhesiveness, elasticity, and cohesiveness) of the emulsion-filled gels (EFG).

Properties *	Temperature (°C)	FC	ECO_10_	ECO_15_	ECO_20_
Hardness (N)	25	0.48 ± 0.01	0.41 ± 0.00	0.35 ± 0.01	0.37 ± 0.01
	37	0.47 ± 0.00	0.41 ± 0.03	0.37 ± 0.01	0.40 ± 0.01
Compressibility (N.mm)	25	2.23 ± 0.05	1.84 ± 0.12	1.67 ± 0.18	1.80 ± 0.01
	37	2.26 ± 0.00	1.90 ± 0.13	1.77 ± 0.10	1.89 ± 0.05
Adhesiveness (N.mm)	25	1.64 ± 0.06	1.50 ± 0.12	1.33 ± 0.15	1.43 ± 0.01
	37	1.67 ± 0.01	1.51 ± 0.31	1.36 ± 0.07	1.52 ± 0.06
Elasticity (mm)	25	0.99 ± 0.00	0.99 ± 0.00	0.99 ± 0.00	1.00 ± 0.00
	37	0.99 ± 0.00	1.00 ± 0.00	0.91 ± 0.00	0.99 ± 0.00
Cohesiveness (Dimensionless)	25	0.89 ± 0.00	0.94 ± 0.04	0.91 ± 0.00	0.93 ± 0.00
	37	0.89 ± 0.01	0.92 ± 0.08	0.88 ± 0.01	0.91 ± 0.01

* The values represent the mean (±standard deviation) of at least five replicates.

**Table 3:** Rheological parameters calculated for emulsion-filled gels (EFG) using Ostwald-de-Waele model.

Parameters *	Temperature (°C)	FC	ECO_15_	ECO_20_
Consistency index (*K*) (Pa.s)	25	78.35 ± 0.96	96.34 ± 8.07	118.70 ± 6.01
	37	79.92 ± 9.88	78.17 ± 1.60	105.93 ± 10.20
Power law index (*n*) (dimensionless)	25	0.38 ± 0.38	0.29 ± 0.02	0.28 ± 0.01
	37	0.37 ± 0.02	0.34 ± 0.00	0.29 ± 0.00
Yield value (Pa)	25	126.05 ± 2.76	0.00 ± 0.00	0.00 ± 0.00
	37	90.48 ± 9.22	40.20 ± 3.36	0.00 ± 0.00
Hysteresis area (Pa/s)	25.0	36,690 ± 4709	589,033 ± 23,122	591,400 ± 10,182
	37.0	45,110 ± 1117	105,640 ± 8146	439,000 ± 54,164

* The values represent the mean (±standard deviation) of at least five replicates.

## Data Availability

Not applicable.
